# P-740. Engagement in the Syphilis Screening Cascade among Pregnant Women in North Carolina

**DOI:** 10.1093/ofid/ofaf695.951

**Published:** 2026-01-11

**Authors:** Stephanie Sweitzer, Erika Samoff, Victoria Mobley, Arlene C Seña

**Affiliations:** University of North Carolina, Chapel Hill, NC; North Carolina Department of Health and Human Services, Raleigh, North Carolina; North Carolina Department of Health and Human Services, Raleigh, North Carolina; Division of Infectious Diseases, Department of Medicine, University of North Carolina at Chapel Hill School of Medicine, Chapel Hill, NC

## Abstract

**Background:**

Congenital syphilis (CS) rates have been rising in the United States, especially in North Carolina (NC). NC public health law requires that healthcare providers screen pregnant women for syphilis during the first prenatal visit, between 28-30 weeks gestation, and at delivery. We describe a syphilis screening cascade for pregnant women accessing care in the University of North Carolina Health System (UNC Health) to identify gaps and opportunities for CS prevention.

**Figure:**
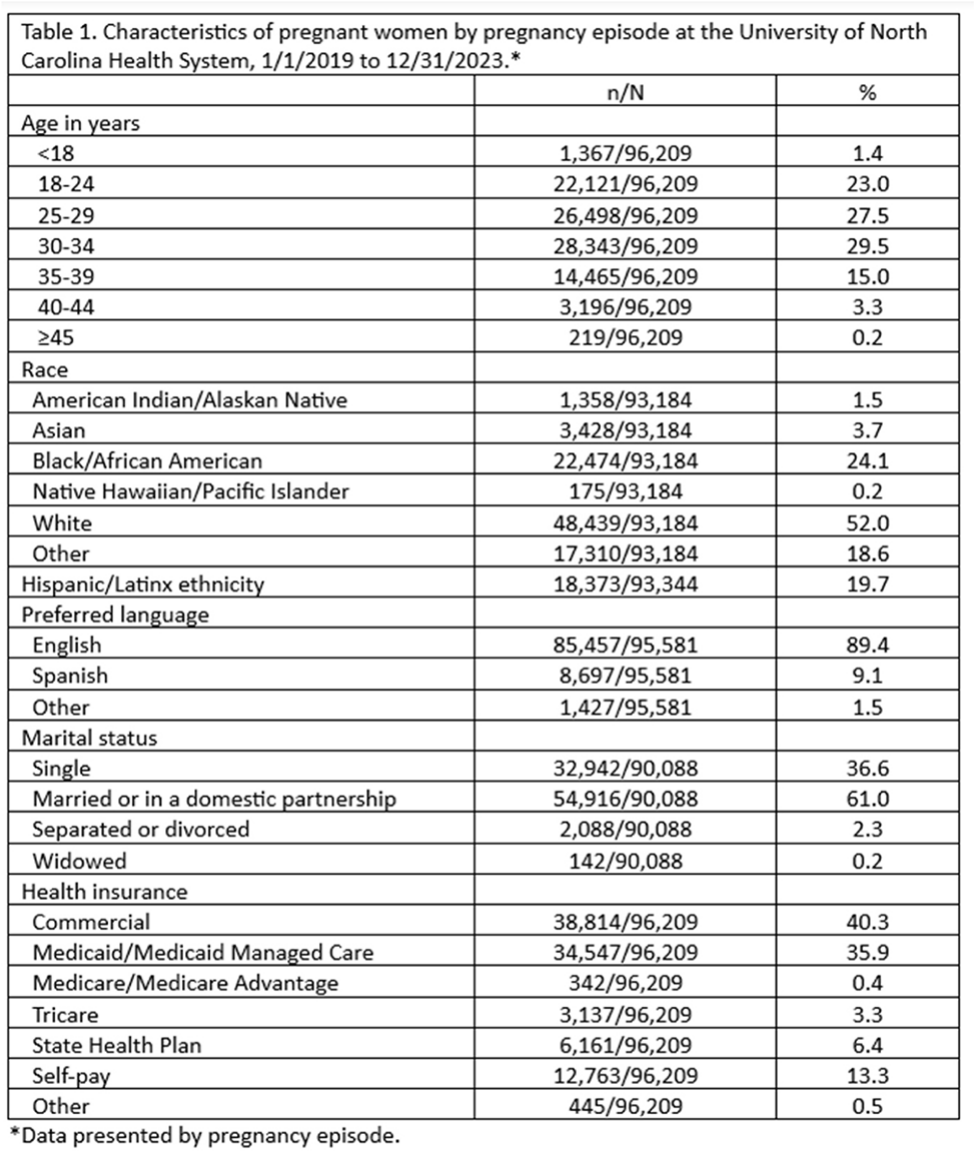

**Figure:**
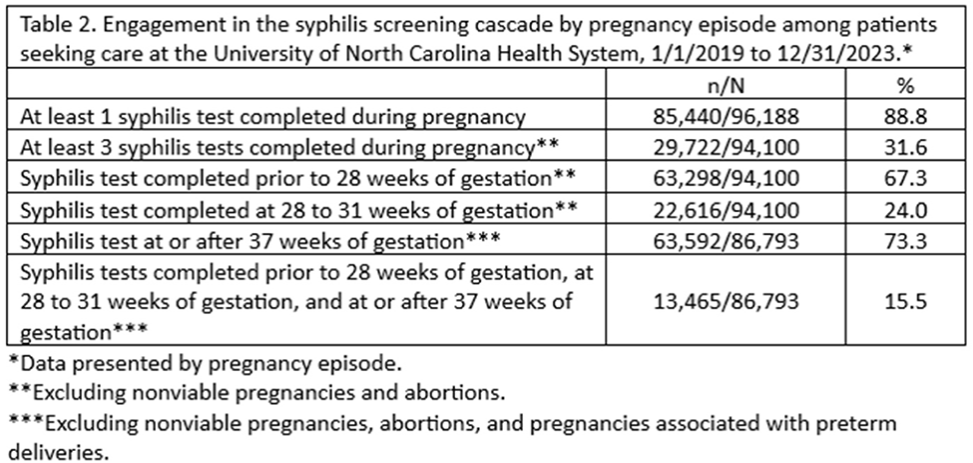

**Methods:**

A retrospective chart review was conducted among patients with an episode of care for pregnancy and/or labor and delivery at UNC Health—a major provider of healthcare services across NC--from 1/1/2019 to 12/31/2023. Patients with estimated delivery dates (EDDs) on/after 12/01/2023 were excluded to allow for sufficient follow up time. Data abstracted from the electronic health record included sociodemographic data, EDDs, care setting, syphilis test results, and birth outcomes. A testing cascade was constructed using syphilis test dates and EDDs. Associations between sociodemographic variables and syphilis testing were explored through univariate regression.

**Figure:**
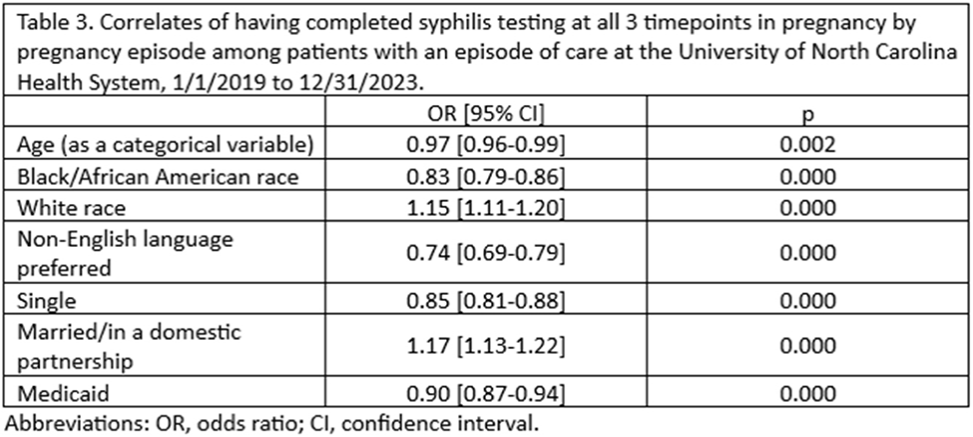

**Figure:**
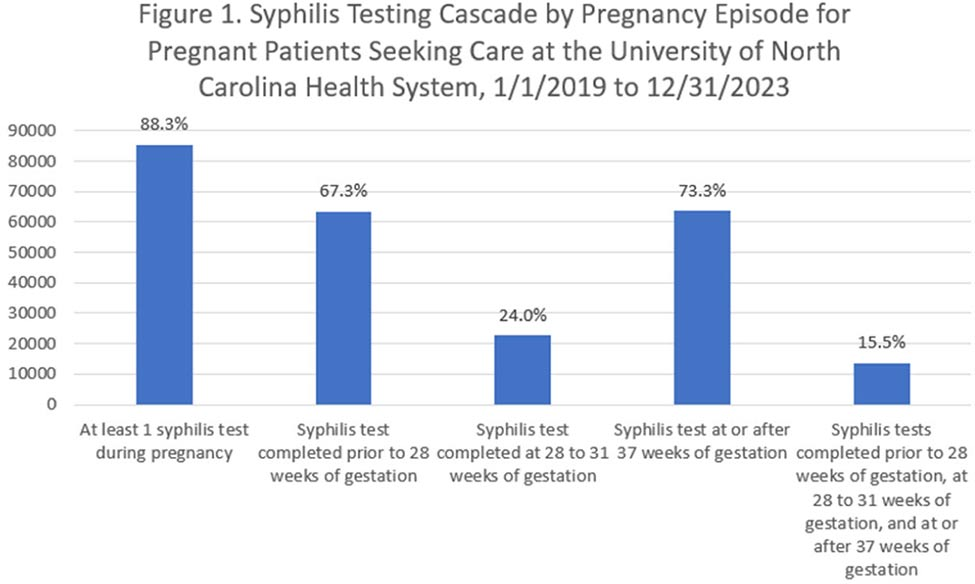

**Results:**

Between 1/1/2019 and 12/31/2023, there were 96,209 pregnancy episodes identified for 81,824 unique women. The majority of pregnancy episodes (89%) included at least one syphilis test. Excluding nonviable pregnancies and abortions, syphilis testing was conducted prior to 28 weeks of gestation in 67% of pregnancy episodes, between 28 and 31 weeks of gestation in 24%, and at or after 37 weeks in 73%. Only 16% of pregnancy episodes had syphilis testing conducted at all three timepoints. Women who were Black/African American, those on Medicaid, and those who required a translator for healthcare communication were less likely to be screened for syphilis at all three timepoints.

**Conclusion:**

Completion of syphilis testing at all three relevant timepoints in pregnancy was low, which could be due to barriers in accessing consistent prenatal care throughout pregnancy or limited provider knowledge of syphilis testing guidelines. Gaps in CS prevention are more pronounced among certain patient populations, suggesting a need for structural interventions within healthcare settings to assure appropriate syphilis screening for all pregnant patients.

**Disclosures:**

Arlene C. Seña, MD, MPH, American Sexually Transmitted Diseases Association: Board Member|University of Alabama at Birmingham: Honoraria|UpToDate: Advisor/Consultant

